# PIN-FORMED1 polarity in the plant shoot epidermis is insensitive to the polarity of neighboring cells

**DOI:** 10.1016/j.isci.2022.105062

**Published:** 2022-09-05

**Authors:** Abdul Kareem, Neha Bhatia, Carolyn Ohno, Marcus G. Heisler

**Affiliations:** 1 School of Life and Environmental Sciences, University of Sydney, Sydney, NSW 2006, Australia; 2 European Molecular Biology Laboratory, Heidelberg 69117, Germany

**Keywords:** Biological sciences, Plant biology, Plant morphology, Plant development

## Abstract

At the *Arabidopsis* shoot apex, epidermal cells are planar-polarized along an axis marked by the asymmetric localization patterns of several proteins including PIN-FORMED1 (PIN1), which facilitates the directional efflux of the plant hormone auxin to pattern phyllotaxis. While PIN1 polarity is known to be regulated non-cell autonomously via the MONOPTEROS (MP) transcription factor, how this occurs has not been determined. Here, we use mosaic expression of the serine threonine kinase PINOID (PID) to test whether PIN1 polarizes according to the polarity of neighboring cells. Our findings reveal that PIN1 is insensitive to the polarity of PIN1 in neighboring cells arguing against auxin flux or extracellular auxin concentrations acting as a polarity cue, in contrast to previous model proposals.

## Highlights

• Epidermal PID expression promotes apical and convergent PIN1 polarity in the shoot
• PID-induced polarization of PIN1 does not depend on neighboring cell polarity

## Graphical abstract

**Figure:**
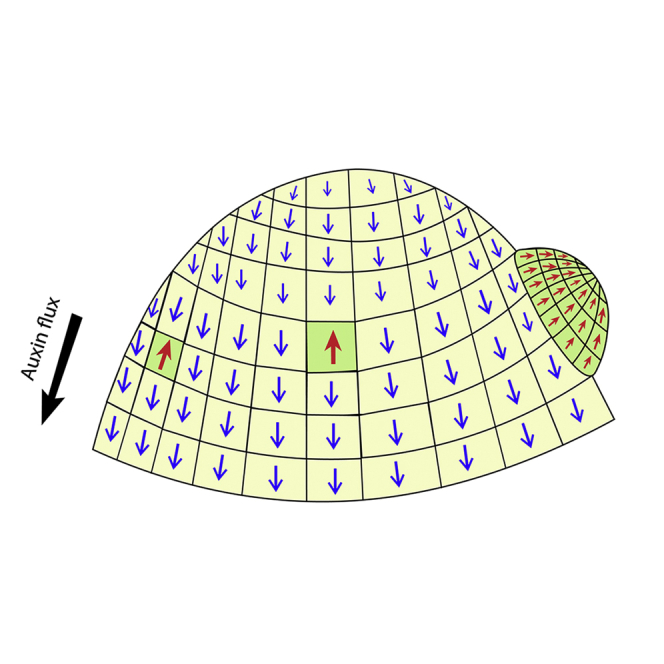

## Introduction

Unlike animals, plants typically generate new lateral organs throughout post-embryonic development, often in a periodic fashion. The spatial organization of these organs, also called phyllotaxis, has been a focus of intense interest for biologists, mathematicians, and physicists alike for many years (Jean and Barabé, 1998). Plant organogenesis is triggered by the plant hormone auxin and in the shoot, auxin is concentrated at organ initiation sites through a polar auxin transport system that depends on the membrane-bound auxin efflux carrier PIN-FORMED1 (PIN1) (Kuhlemeier and Reinhardt, 2001; Okada et al., 1991; Reinhardt et al., 2000). PIN1 directs auxin flux according to its asymmetric or polar localization, and in meristem epidermal cells at a supracellular scale, PIN1 polarity forms convergence patterns oriented toward both organ initiation sites as well as the shoot apex (Galvan-Ampudia et al., 2020; Heisler et al., 2005; Mansfield et al., 2018; Reinhardt et al., 2003).

Given that polarity convergence patterns dictate where auxin accumulates and therefore where new organs form, in order to understand plant phyllotaxis, it is necessary to understand how such polarity patterns are generated. Through computational modeling, a number of studies have shown that feedback between auxin and the polarity of its transporter protein PIN1 can account for PIN1 convergence patterns (Abley et al., 2016; Cieslak et al., 2015; Hartmann et al., 2019; Heisler et al., 2010; Heisler and Jonsson, 2006; Smith et al., 2006; Stoma et al., 2008). These models differ in the way auxin is proposed to provide polarity information. For instance, “up the gradient” models propose that cells polarize their PIN1 toward neighboring cells in proportion to the relative internal auxin concentrations (Jonsson et al., 2006; Smith et al., 2006) with mechanical signals acting to mediate cell-cell communication (Heisler et al., 2010). Another set of models propose auxin regulates PIN1 polarity via its net flux across the plasma membrane or as an extracellular coupling factor that regulates intracellular polarity partitioning (Abley et al., 2013, 2016; Stoma et al., 2008). A central prediction of the latter proposals is that the polarity of PIN1 is sensitive to the polarity of PIN1 in neighboring cells, assuming these cells are transporting auxin (Abley et al., 2013, 2016).

Although the above-mentioned classes of models are relatively well established in the literature, few studies have tested their assumptions experimentally. In an effort to test the “up the gradient” model, Bhatia et al. engineered differences in auxin signaling between neighboring cells by inducing clonal expression of the auxin response factor MONOPTEROS (MP) in *mp* mutants. These experiments demonstrated that local differences in auxin signaling between cells can indeed act as a polarity cue, as “up the gradient” models would predict (Bhatia et al., 2016). However, the observed reorientation of PIN1 polarity toward MP expressing cells can also be explained by flux-based and auxin indirect coupling models if local MP expression induces expression of auxin influx carriers and auxin degradation or conjugation enzymes, such that there is net auxin influx (Abley et al., 2016). Furthermore, it is possible to envision a scenario in which mechanics plays a role to orient a polarity axis while flux determines PIN1-mediated efflux direction (Marconi et al., 2021). Here, we test indirect coupling models and models based on auxin flux by assessing the sensitivity of PIN1 polarity to the polarity of PIN1 in neighboring cells in the *Arabidopsis* shoot using mosaic expression of the serine threonine kinase PINOID (PID).

## Results

**Figure 1:**
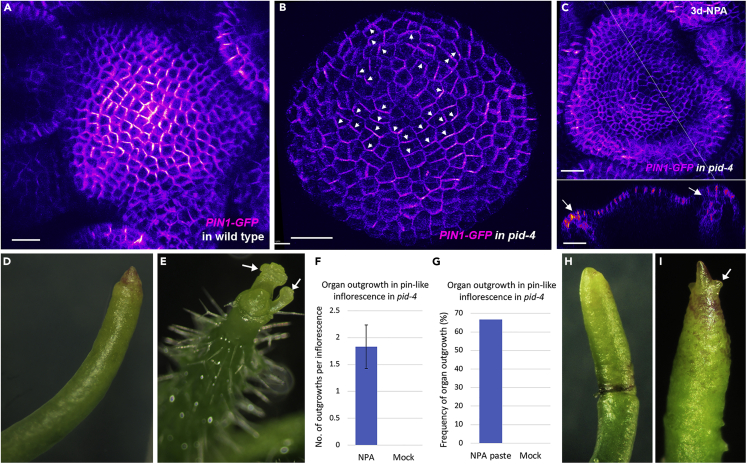
Basally localized PIN1 in *pid* mutant meristem likely transports auxin basally (A) *PIN1::PIN1-GFP* expression (magenta) in wild type inflorescence meristem. (B) *PIN1::PIN1-GFP* expression in *pid-4* mutant inflorescence meristem. Basal PIN1 polarity away from meristem center marked by arrowhead. (C) *pid-4 PIN1-GFP* inflorescence meristem forming floral organ tissue 3 days after 10μM NPA treatment. Lower image shows longitudinal optical section of the meristem corresponding to line in top image. Arrows indicate organ primordia. (D) Mock-treated *pid-4 PIN1-GFP* inflorescence. (E) *pid-4 PIN1-GFP* apex treated with 10μM NPA for 7 days. Arrows indicate floral primordia. (F) Graph showing number of organ primordia produced from the *pid-4* mutant apex after NPA and mock treatments (n = 12). Error bar represents SE of mean. (G) Graph showing frequency of organ outgrowth from the pin-like inflorescences of *pid-4* mutant after local application of NPA in lanoline paste (n=9) and mock treatments (n=5). (H) Mock treated *pid-4 PIN1-GFP*inflorescence. (I) *pid-4 PIN1-GFP* apex treated with 10µM NPA in lanoline paste. Arrow indicates organ primordia. Scale bar = 100 μm (A), 20 μm (B–C).

The PID serine threonine kinase is known to directly phosphorylate PIN1 (Christensen et al., 2000; Michniewicz et al., 2007) and its activity is both necessary and sufficient to promote apical polarization of PIN1 in the root and embryo (Friml et al., 2004). In the shoot, PID also promotes a convergent or apical polarity since in *pid* mutants, PIN1 is predominantly polarized in a divergent pattern away from the apex and toward the root (Friml et al., 2004) (Figures 1A and 1B). In addition to promoting apical polarization, PID activity also enables PIN1 localization to respond to mechanical signals (Friml et al., 2004; Heisler et al., 2010), such as those involved in organ formation (Hamant et al., 2008). We note that besides regulating PIN1 polarity, PID has been implicated in activating PIN1-mediated auxin efflux, as assessed in heterologous assays using Xenopus oocytes (Zourelidou et al., 2014). However, genetic data strongly indicate that *in planta*, PIN1 retains efflux activity in the absence of PID, possibly due to the redundant activities of PID2, WAG1, and WAG2 (Cheng et al., 2008; Reinhardt et al., 2003).

### Basally localized PIN1 in *pid* mutant meristem likely transports auxin basally

Auxin transcriptional responses, as detected by the DR5 reporter, are absent in the *pid* mutant apex Galvan-Ampudia et al., 2020), as might be expected if PIN1 transports auxin basally due to its basal polarity. To further investigate this possibility, we grew *pid-4* plants on 10 μM n-naphthylphthalamic acid (NPA) to see whether reductions in auxin transport could alter the flowerless *pid-4* phenotype and found that flower formation was partially rescued (Figures 1C–1F). While 75% of NPA-treated pin-like inflorescences produced an average of 1.8 flowers per inflorescence (n = −12), mock-treated inflorescences did not make any flowers (n = 12) (Figure 1D). This was further confirmed by the local application of NPA paste on pin-like inflorescences where in 66.6% NPA-treated inflorescences developed outgrowth (n = 9) compared to none developing after treatment with control paste (n = 5) (Figure 1G–I). These data support the proposal that in *pid-4* mutants, basally localized PIN1 transports auxin basally, thereby depleting auxin from the meristem and causing a flowerless phenotype, although the involvement of additional auxin transporters cannot be ruled out.

### Epidermal PID rescues organ formation and acts cell-autonomously to reorient PIN1 polarity

**Figure 2:**
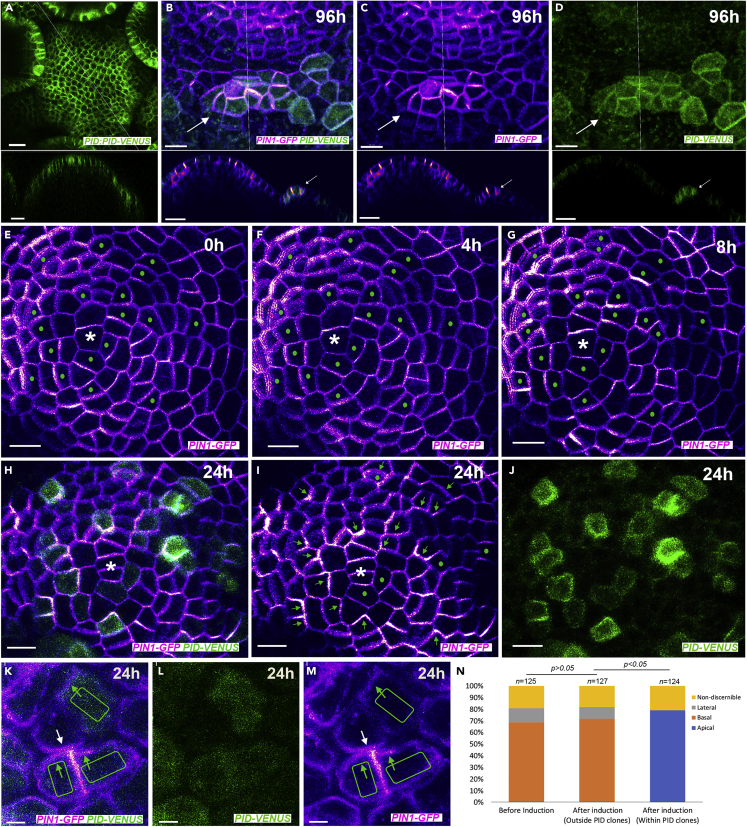
PID promotes changes in PIN1 polarity irrespective of initial or neighboring cell polarities (A) Expression pattern of *PID::PID-2V* (green) in wild-type inflorescence meristem. Lower image represents longitudinal optical section corresponding to line in the upper image. (B–D) Organ primordium (arrow) associated PIN1 convergence (magenta) co-localized with clonal PID expression (green) 96 h after clone induction. Lower images in B–D represent longitudinal optical sections corresponding to line in upper images. PID-2V and PIN1-GFP (B), PIN1-GFP (C), and PID-2V (D). (E–J) Time-lapse images of the *pid-4* meristem before (E) and after (F–J) induction of *PID-2V* (green) expressing clones showing changes in *PIN1-GFP* (magenta) polarity. The time interval of imaging: 0 h (E), 4 h (F), 8 h (G), and 24 h (H–J). Note apical shift in PIN1-GFP polarity (green arrows in I) in cells expressing PID-2V (green dots in E–J). Green dots in (I) mark cells with unclear polarity. Asterisk marks middle of the meristem. (K–M) Magnified high-resolution images of plasmolyzed *pid-4* mutant meristem showing opposing PIN1-GFP (magenta) polarities (green and white arrows in (K) and (M)) in adjacent cells due to differential PID-2V expression (green) 24h after induction. PID-2V and PIN1-GFP (K), PID-2V (L), and PIN1-GFP (M). (N) Quantification of the shift in PIN1 polarity in *pid-4* meristem after the induction of PID-2V (n = 124). Scale bar = 100 μm (A), 10 μm (B–D) upper panels, 15 μm (B–D) lower panels, 10 μm (E–J), 2 μm (K–M).

Given PID is present throughout the shoot epidermis, albeit at higher levels in boundary regions (Figure 2A), we sought to restore local PID expression in *pid* mutants to enable proper polarization of PIN1 and allow us to assess whether PIN1 polarizes according to the prevailing pre-existing polarity of the tissue. Using an inducible system for Cre-lox recombinase-mediated recombination, we generated epidermal clones of cells constitutively expressing PID fused to two copies of VENUS (PID-2V) in *pid* mutant shoot apical meristems (SAMs). We found epidermal PID expression to be sufficient for rescuing organ formation since when large sectors expressing PID-2V were generated, organs formed that were marked by PID-2V expression (Figures S1A–S1D). Closer examination also revealed local PID-2V expression associated with the formation of polarity convergence patterns at the SAM periphery (Figures 2B–2D and Video S1).

Video S1. PIN1 polarity convergence promoted by clonal PID expression, related to Figure 2 Animated volume rendering of primordium depicted in Figures 2B–2D

Next, we examined PIN1 signal within membrane-localized PID-2V marked cells over time, from when PID-2V signal first became detectible 6–8 h after clone induction. We found a distinct basal to apical shift in PIN1 polarity in PID-2V marked cells, even when such cells were surrounded by PID negative cells that were polarized basally (Figures 2E–2J, 2N, S1E–S1R, and Video S2). We also found that PID positive clones did not influence the polarity of PIN1 in mutant neighboring cells, which remained basal (Figures 2K–M, S1S–S1U), consistent with previous findings that PID is required for changes to PIN1 polarity in the shoot epidermis (Heisler et al., 2010).

Video S2. PIN1 polarity changes over time in response to PID expression, related to Figure 2 Animated series of four time points depicted in Figures 2E-2J showing shift in PIN1-GFP (magenta) polarity from basal to apical in PID-2V (green) expressing cells in *pid-4* mutant meristem

## Discussion

While our experiments do not rule out the formal possibility that PID may be required in neighboring cells for a PID positive cell to perceive neighboring cell PIN1 polarity, the simplest conclusion based on our results is that PIN1 polarity in shoot epidermal cells does not depend directly on the polarity of PIN1 in neighboring cells. This finding contrasts with predictions from flux-based or indirect coupling models using auxin acting as the coupling agent (Abley et al., 2013, 2016; Stoma et al., 2008) and instead, favors alternative models in which auxin plays a role in orienting PIN1 independent of its transport, for instance, via auxin-induced changes to mechanical stresses in the cell wall (Bhatia et al., 2016; Heisler et al., 2010). However, while mechanical signals may account for convergent polarity patterns (including toward the shoot apex), they are unlikely to be capable of aligning plant cell polarities over large distances, suggesting additional mechanisms may be involved (Bhatia and Heisler, 2018). Furthermore, our conclusions are relevant only to the shoot epidermis and not other tissues where PIN1 can orient in distinct patterns. Gaining a full understanding of how plant cells orient and coordinate polarity remains a significant challenge for the future.

### Limitations of the study

In this study, we focus on the shoot epidermis of *Arabidopsis thaliana* and find that cells expressing PID do not tend to align their polarity with neighboring *pid* mutant cells and vice versa. Hence, our study is limited to a particular tissue, the *Arabidopsis* shoot epidermis. Our experiments also do not rule out the possibility that PID may be required in neighboring cells for PID positive cells to perceive neighboring cell PIN1 polarity.

## STAR★Methods

### Key resources table

**Table:** 

REAGENT or RESOURCE	SOURCE	IDENTIFIER
**Bacterial and virus strains**		

Agrobacterium tumefaciens C58C1	N/A	N/A
DH5α competent cells	NEB	CAT#C2987I

**Chemicals, peptides, and recombinant proteins**		

Murashige and Skoog Medium (MS)	Sigma	Cat#M5524
2-(MN-morpholino)- ethane sulfonic acid (MES)	Sigma	Cat#M2933
Bacto agar	BD	Cat#214010
MS vitamins	Sigma	Cat#M3900
Dexamethasone (DEX)	Sigma	Cat#D4902
1-N-Naphthylphthalamic acid (NPA)	Sigma	Cat#33371
Dimethyl sulfoxide (DMSO)	Sigma	Cat#276855
Paraformaldehyde	Sigma	Cat# 158,127
Tween 20	Sigma	Cat# P1379
KCl	Sigma	Cat#P9541
NaCl	Sigma	Cat#S3014
Na_2_HPO_4_	ChemSupply	Cat# SA026
KH_2_PO_4_	ChemSupply	Cat# PA009
Sucrose	ChemSupply	Cat# SA030
Gentamycin sulfate	Duchefa	G0124.0025
**Spectinomycin HCl pentahydrate**	Duchefa	S0188.0025
Rifampicin	Duchefa	R0146.0005
Glufosinate-ammonium	Sigma	Cat#45520
Primestar GXL DNA Polymerase	Takara	Cat#R050
In-Fusion	Takara	Cat#638911
SfiI	NEB	Cat#R0123S
BamHI	NEB	Cat#R3136S
BglII	NEB	Cat#R0144S
LR clonase	Thermo Fisher	Cat#11791020

**Experimental models: Organisms/strains**		

*Arabidopsis thaliana* Landsberg erecta (Ler)	N/A	N/A
Arabidopsis *pid-4* mutant *(*Ler*)*	(Bennett et al., 1995)	N/A
Arabidopsis PIN1::PIN1-GFP, PID::PID-VENUS	(Heisler et al., 2005) (Michniewicz et al., 2007)	N/A
Arabidopsis *pid-4 PIN1::PIN1-GFP*	This study	N/A
Arabidopsis *pid-4 PIN1::PIN1-GFP + pML1::CRE-GR* + *pUBQ10::lox-GUS-lox-PID-2XVENUS*	This study	N/A

**Oligonucleotides**		

*Primers (see*experimental model and subject details*)*	This study	N/A

**Recombinant DNA**		

*transfer DNA vector BGW*	(Karimi et al., 2002)	N/A

**Software and algorithms**		

*Leica application suite AF*	Leica	www.leica-microsystems.com
Imaris 9.1.2	Bit-plane	https://imaris.oxinst.com
ImageJ FIJI	Open-source software	https://fiji.sc
Adobe Photoshop 2020	Adobe	www.adobe.com/products/photoshop.html
SPSS statistics	IBM	www.ibm.com/products/spss-statistics

### Resource availability

#### Lead contact

Further information and requests for resources and reagents should be directed to and will be fulfilled by the lead contact, Marcus G. Heisler (marcus.heisler@sydney.edu.au).

#### Materials availability

There are no restrictions to the availability of newly generated materials in this study.

### Experimental model and subject details

#### Plant materials and growth conditions

*A. thaliana* ecotype *Landsberg erecta* (*Ler*) was used as the wild type in this study. The *pid-4* mutant *(Ler)* allele was previously described (Bennett et al., 1995). Transgenic lines used in this study include *pid-4* mutant transformed with *pPIN1::PIN1-GFP* reporter and *pid-4 PIN1::PIN1-GFP* transformed with *pML1::CRE-GR* + *pUBQ10::lox-GUS-lox-PID-2XVENUS.* Seeds were germinated and grown on growth medium (GM) containing 1X Murashige and Skoog (MS) basal salt mixture (Sigma M5524), 1% sucrose, 0.5% MES 2-(MN-morpholino)- ethane sulfonic acid (Sigma M2933), 0.8% Bacto Agar (BD) and 1% MS vitamins (Sigma M3900). pH was adjusted to 5.7 with 1M KOH. Plants were grown at 22°C under continuous light. For imaging of wild type inflorescence meristems, plants were grown on soil at 18°C in short day conditions (16h/8h).

### Method details

#### Construction of reporters and transgenic plants

For mosaic analyses using the CRE/Lox system, stable transgenic lines harboring a template for sectoring (UBQ10p:lox spacer lox:PID-2XVENUS) and a dexamethasone-inducible CRE Recombinase (ML1p:CRE-GR) were used. To generate UBQ10p:lox spacer lox:PID-2XVENUS, first a 4.6 kb SfiI-BamHI fragment from UBQ10p:lox spacer lox:MP-VENUS (Bhatia et al., 2016) was cloned upstream of a 9X alanine linker followed by 2 tandem copies of VENUS and OCS terminator. PID cDNA sequence was amplified with primer set 121 (5′GGATCCAACAATGGCATGTTACGAGAATCAG-3′) and 122 (5′GGATCCCCAAAGTAATCGAACGCC-3′) and then cloned BamHI fragment as a translational fusion to 2XVENUS to create UBQ10p:lox spacer lox:PID-2XVENUS. CRE-GR (Bhatia et al., 2016) was cloned as a BglII fragment downstream 3.38 kb of ATML1 (At4g21750) 5′-regulatory sequences amplified with primer set 0708H3f (5′AAGCTTATCAAAGAAAAAACAAGAACAAAACG-3′) and 0708Br (5′GGATCCACACCCGGTGGATTCAGGGAGTTTC-3′) to generate ML1p:CRE-GR. Finally, the UBQ10p:lox spacer lox:PID-2XVENUS, and ML1p:CRE-GR were combined in transfer DNA vector BGW (Karimi et al., 2002) by Gateway technology (Invitrogen). The constructs were further transformed into agrobacterium strain C58C1 by electroporation and then finally transformed into Arabidopsis by floral dipping.

#### NPA and DEX treatments

For NPA treatment, *pid-4 PIN1-GFP* seedlings were grown on GM agar plate until pin-like inflorescence meristem formation. The entire seedlings were then transferred onto the GM agar plate supplemented with 10μM NPA. Plants were then grown continuously on NPA containing medium and imaged under confocal and/or brightfield microscope for 3–7 days. For mock treatment, the plants were shifted from GM agar plate to GM agar with equivalent volume of DMSO. For local NPA treatment, lanoline paste carrying 10µM NPA was administered at the periphery of pin-like inflorescence meristem and then the organ outgrowth was followed after 4–7 days.

To induce PID-2XVENUS sectors in epidermis, *pid* mutants harboring *ML1p::CRE-GR + UBQ10p::lox spacer lox::PID-2XVENUS* were grown on GM agar plate until the emergence of flowerless dome. Then 10–20μL of 10μM DEX in sterile water (10mM stock, dissolved in absolute ethanol) were directly applied on the dome meristem and were imaged at different time interval (until 24 h or 96 h). For every experiment, *pid* mutants were imaged before and after induction.

#### Sample preparation for confocal live imaging

For Figures 1 and 2A, wild-type inflorescence meristem was dissected and mounted on GM agar plate as previously described (Heisler and Ohno, 2014). For *pid* mutant meristem imaging, the leaves of the mutant plant covering the dome meristem were dissected away under water and then mounted the whole plant with roots on GM agar plate. Prior imaging, the mounted plants were kept submerged under sterile water for 30 min to halt meristem growth. For high resolution images (for Figures 2K–2M), meristem was briefly fixed in 4% paraformaldehyde as described previously (Heisler et al., 2005) and then plasmolyzed in 1M Sucrose for 1h. The plasmolyzed meristem was imaged after mounting the meristem on GM agar plate filled with sucrose solution.

#### Settings for confocal live imaging and time-lapse imaging

Confocal live imaging was performed on a Leica TCS-SP5 upright laser scanning confocal microscope with hybrid detectors (HyDs) using a 25X water objective (N.A 0.95) or 63X objective (N.A 1.20). Either 512x512 or 1024x1024 (for high resolution images) pixel format was used. Bidirectional scan was set with a scan speed of 400Hz or 200Hz. Line averaging used was 2 or 3. The thickness of the optical sections was 1μm.

Argon laser was used for both GFP and VENUS. GFP was excited using 488nm laser and emission window was set to 493–512nm. VENUS was excited with 514 nm laser and detected using a 520–560 nm window. Pinhole was adjusted depending on the fluorescence brightness to prevent signal saturation or bleaching. Smart gain was set to 100%. Sequential scan mode with switching in between-frames was used for imaging GFP and VENUS together.

To monitor the shift in PIN1 polarities with respect to PID clones, a time lapse imaging experiment was performed on *pid* mutant harboring *ML1p::CRE-GR + UBQ10p::lox spacer lox::PID-2XVENUS*. The time lapse imaging was followed at every 1 h or 2 h interval for a duration of 12 h or 24 h. PIN1-GFP expression was monitored at all time intervals, but PID-2XVENUS expression was monitored only at 2 or 3 intervals to minimize photo-toxicity. After every scan, the plants were quickly placed back under light after removing the water.

#### Image analysis and data processing

The images were analyzed using Imaris 9.1.2 (bit-plane), or ImageJ (FIJI, https://fiji.sc). The images were annotated and arranged in Adobe Photoshop (2020). PIN1 polarity assessments were made according to the presence of arcs of GFP signal extending beyond cell junctions and around cell corners.

### Quantification and statistical analysis

Statistical analyses were performed using Excel or SPSS Statistics. The χ2 test was conducted for analysis of statistical significance (Figure 2N). Statistical details of the experiments can be found in the figure legends.

## Data Availability

This study did not generate new resource data or code.
