# Supplementary material for: PIN-FORMED1 polarity in the plant shoot epidermis is insensitive to the polarity of neighboring cells
Source: iScience. 2022 Sep 5;25(10):105062. doi: 10.1016/j.isci.2022.105062 (PMC9494258; doi:10.1016/j.isci.2022.105062)
Supplement: Document S1. Figures S1 [file mmc1.pdf]

iScience, Volume *25*

Supplemental information

PIN-FORMED1 polarity in the plant shoot epidermis is insensitive to the polarity of neighboring cells

Abdul Kareem, Neha Bhatia, Carolyn Ohno, and Marcus G. Heisler

Supplemental information

PIN-FORMED1 polarity in the plant shoot epidermis is insensitive to the polarity of neighbouring cells

Abdul Kareem, Neha Bhatia, Carolyn Ohno & Marcus G. Heisler

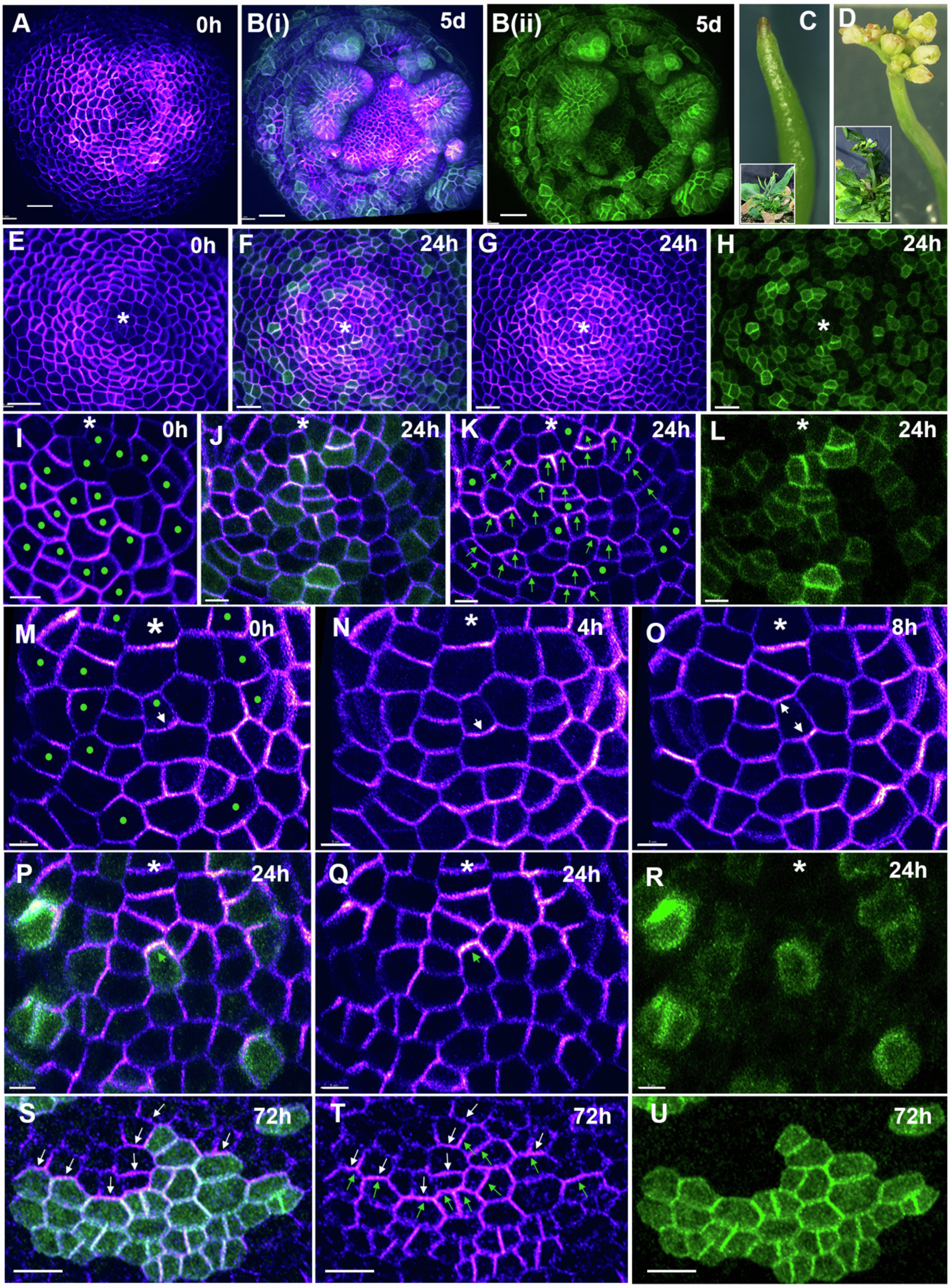

Figure S1. PIN1 polarity in response to PID clones, Related to Figure 2.

(A) *pid-4* meristem expressing PIN1-GFP (magenta) before induction of PID-2V. (B-i) Same *pid-4* meristem expressing PIN1-GFP (magenta) as in (A) 5 days after clonal induction of PID-2V

(green). Note developing organs marked by large sectors of PID-2V expression. (B-ii), Same as B-i showing PID-2V (green) alone. (C) pin-like structure of uninduced *pid-4 PIN1-GFP* inflorescence. (D) Inflorescence of *pid-4 PIN1-GFP* rescued by clonal expression of PID-2V after 1 week. (E-R) Images of the *pid-4* meristem before (E,I,M) and after (F-H, J-L, N-R) induction of *PID-2V* (green) expressing clones showing changes in *PIN1-GFP* (magenta) polarity. (I-L) Magnified images of the same meristem in E-H. (M-R), Magnified high resolution time-series images of the same meristem from Figure 2 E-J. Note apical shift in PIN1-GFP polarity (green arrows in K,P, and Q) in cells expressing PID-2V (green dots in I,M). Green dots in K mark cells with undiscernible polarity. Note a gradual shift in PIN1-GFP (magenta) polarity from basal (M,N) to bipolar (O) to apical (P,Q) over time after induction of PID-2V (green). Arrows indicate observed polarity, i.e., downward= basal, upward= apical, two arrows in opposite directions = bipolarity. Asterisk marks middle of the meristem. (S-U) Flank of a *pid-4* shoot apex showing opposing PIN1-GFP (magenta) polarities (green and white arrows in S and T) in adjacent cells due to differential PID-2V expression (green) (S,U) 3days after induction. Scale bar = 30 μm (A-B), 20 μm (E-H), 7 μm (I-L), 5 μm (M-R), 20 μm (S-U).
